# Adult non-urethral complications after hypospadias repair in childhood: presentation, treatment and outcomes

**DOI:** 10.3389/fendo.2023.1184948

**Published:** 2023-06-08

**Authors:** Borko Stojanovic, Marta Bizic, Marko Bencic, Miroslav L. Djordjevic

**Affiliations:** ^1^ Belgrade Center for Urogenital Reconstructive Surgery, Belgrade, Serbia; ^2^ School of Medicine, University of Belgrade, Belgrade, Serbia; ^3^ Department of Urology, Icahn School of Medicine at Mount Sinai, New York, NY, United States

**Keywords:** hypospadias, penile curvature, trapped penis, complications, reconstruction

## Abstract

**Purpose:**

This study aimed to evaluate a group of adult patients with non-urethral complications after hypospadias repair in childhood, their surgical treatment, and outcomes.

**Materials and methods:**

We analyzed 97 patients, mean age 22.5 years, who were treated in our center between January 2009 and December 2020, for non-urethral complications after previous hypospadias repair in childhood. Non-urethral complications were defined as glans deformity, residual curvature and trapped penis due to insufficient penile skin. A radical surgical approach was used to correct all deformities, in a one-stage or a two-stage procedure. A successful outcome was defined as a straight penis with good length, anatomically regular glans, and cosmetically acceptable appearance, without need for additional surgeries. Sexual function was evaluated using International Index of Erectile Function.

**Results:**

Mean follow-up was 75 months (ranged from 24 to 168 months). One-stage and two-stage repair were performed in 85.5% and 14.5% of cases, respectively. A higher success rate was noted after one-stage repair (94% compared to 86%). Complications included four cases of penile curvature with late onset, one case of glans dehiscence and partial skin necrosis. Erectile dysfunction was determined in 24% of patients.

**Discussion:**

Non-urethral complications may occur many years after primary hypospadias repair, with a strong impact on the quality of life. Treatment is individualized and usually involves a radical surgical approach to correct all associated deformities and to achieve successful cosmetic and psychosexual outcomes.

## Introduction

Hypospadias repair still presents a great challenge for pediatric urologists, despite various new techniques and a better understanding of outcome measures. The main goals of treatment are regular voiding, good aesthetic appearance, and normal sexual function in adulthood, accomplished in a one-stage surgery. Surgery outside the optimal age bracket (after the age of 18 months), severe form of hypospadias and multiple operations, are all defined as risk factors for complications and poor psychological outcome (1, 2). Although most complications are observed within the first year after hypospadias repair, lately there have been many reports of complications that occur even decades after primary repair (3, 4). Complications such as sexual dysfunction and psychosexual difficulties become obvious when patients are sexually active. Many theories have been proposed to explain these late complications, but almost exclusively focusing on urethral complications (stricture, fistula, diverticulum). However, there is strong evidence that a satisfactory cosmetic result of hypospadias surgery is of utmost importance for future psychosexual health (4). Even 30 years ago, Bracka evaluated a large cohort of 213 patients after hypospadias repair, and more then 70% reported that normal appearance of the penis is at least as important as having a functionally appropriate penis (5). Many adult patients report the small size of the penis, which is trapped by scar tissue after previous hypospadias surgeries, as the main cause of their dissatisfaction (6). That is why non-urethral complications after hypospadias repair, especially the ones that present during or after sexual maturity, can have devastating consequences on a young man’s life.

Another issue is how to manage these late-presenting complications. Hypospadias repair in adults carries a significantly higher risk than surgery in childhood owing to differences in tissue quality and wound healing, as well as susceptibility to infection (7). Repeated surgery in failed hypospadias carries the additional risk of insufficient healthy tissue and poor vascular supply, presenting an ultimate challenge for a reconstructive urologist. We present a group of adult patients who presented with non-urethral complications after hypospadias repair during childhood, along with their classification, surgical treatment, and outcomes.

## Materials and methods

We retrospectively reviewed 97 patients, aged 17 to 41 years (mean 22.5), who were treated in our center between January 2009 and December 2020, for non-urethral complications after previous hypospadias repair in childhood. Most patients (95) were initially operated on elsewhere. A complete history of previous repair was not available in 12 cases, and we used patients’ self-reports regarding the timing of surgery, type of anomaly (mild or severe) and number of repairs.

Non-urethral complications presented as glans deformity, residual penile curvature and/or trapped penis. Some patients presented with two or all three complications, defined as cripple penis, or with co-existing urethral complications. A radical surgical approach was used in all cases to correct all deformities in a one-stage or a two-stage procedure.

### Glans reconstruction

Glans reconstruction was performed by mobilization of the wide glans wings using sharp dissection, to the top of the cavernosal body, and their joining in two layers, using interrupted sutures. In cases of small and deformed glans, a “double-faced” skin flap was used to increase glans volume.

### Penile curvature repair

Artificial or pharmacological erection (induced by prostaglandin E1) was used intraoperatively in all cases to evaluate the potential curvature. In severe cases, correction of penile curvature was performed by plication of the tunica albuginea, or grafting technique.

### Trapped penis

Vascularized genital flaps and free skin grafts were used for the reconstruction of the trapped penis because of scarce penile skin. Depending on size of defect and the availability of healthy skin, the penile shaft is released from scars, and covered with skin flaps or grafts.

### Urethral reconstruction

Simultaneous urethral reconstruction was performed using various techniques, in one or two stages. The availability of tissues for reconstruction dictated the approach. The techniques used included urethral mobilization, urethral plate tubularization, combined urethral plate and skin flap, andcombined buccal mucosa graft and hairless skin flap. A suprapubic tube was used for urine derivation for three weeks after surgery, in all patients.

Descriptive statistics were used to evaluate demographic parameters, including the number and timing of previous surgeries, according to the hypospadias type or stages performed. Erectile function was evaluated using the International Index of Erectile Function (IIEF-5), a 5-item self-administered questionnaire scale (8). All patients who reported attempting sexual intercourse in the last six weeks were asked to complete the questionnaire. Categories of erectile dysfunction are defined as: none (≥26), minimal (18–25), moderate (11-17), and severe (≤10).

## Results

The mean age at the first surgery was 4.5 years (ranged from 2 to 10 years), and the mean time following initial repair was 17 years (ranged from 13.5 to 25 years). Distal or midshaft hypospadias were the primary anomalies in 63 patients (65%), while 34 (35%) had severe, proximal hypospadias. The average number of previous surgeries was 1.5, ranging from one to five, and all surgeries were performed during childhood. (**Table 1**)

**Table 1:** Patients’ demographic characteristics in different types of hypospadias.

Form of hypospadias	Number of patients (%)	Mean age at primary surgery (years)	Mean time after primary surgery (years)	Number of previous repairs (mean)
**Distal**	63 (65%)	6	15	1-4 (1.1)
**Proximal**	34 (35%)	2.5	19.5	1-5 (2.3)
**Summary/Mean**	97 (100%)	4.5	17	1.5

Non-urethral complications were classified as glans deformity, residual curvature and trapped penis due to insufficient penile skin and scar tissue; and were repaired in 37, 48 and 33 cases, respectively. Most of these deformities (67%) were late complications of distal hypospadias, and only 33% were related to severe hypospadias. (**Table 2**) Seventeen patients were treated for 2 or more associated complications, including 4 cases defined as a cripple penis. In 25 patients (26%) there was a co-existing urethral complication (7 fistulas, 10 strictures, 4 diverticula, 2 urethral defects, 2 cases of “short urethra”) that required simultaneous repair. (**Table 2**)

**Table 2:** Late complications in different types of hypospadias.

Form of hypospadias	Glans deformity (%)	Residual curvature (%)	Trapped penis (%)	Total non-urethral (%)	Urethral (%)
**Distal**	26 (70%)	39 (81%)	14 (42.5%)	79 (67%)	9 (36%)
**Proximal**	11 (30%)	9 (19%)	19 (57.5%)	39 (33%)	16 (64%)
**Summary**	37 (100%)	48 (100%)	33 (100%)	118 (100%)	25 (100%)

**Figure 1:**
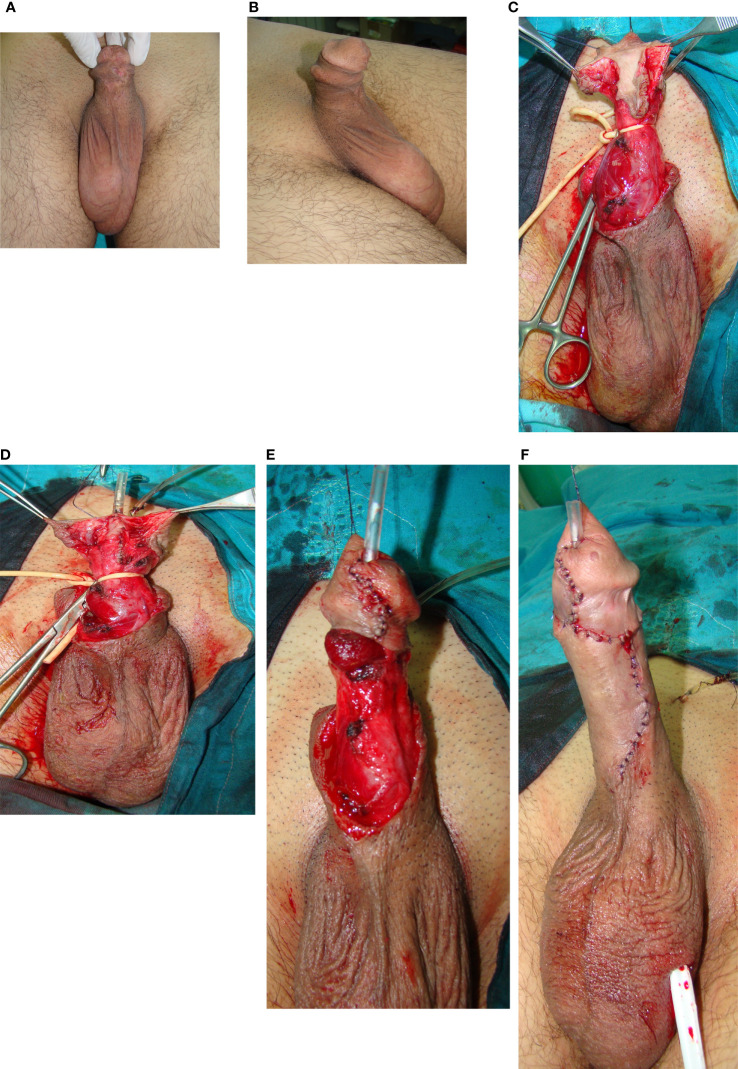
Glans reconstruction with urethroplasty after failed hypospadias surgery in childhood. **(A)** Glans deformity, urethral defect and scarred penile skin are visible. **(B)** Skin deformities - lateral view. **(C)** After degloving, radical glans dissection is done. Urethral plate is mobilized. **(D)** Distal urethroplasty is done by tubularization of urethral plate. **(E)** Glans is closed in two layers to create conically shaped glans. **(F)** Penile skin reconstruction is done.

**Figure 2:**
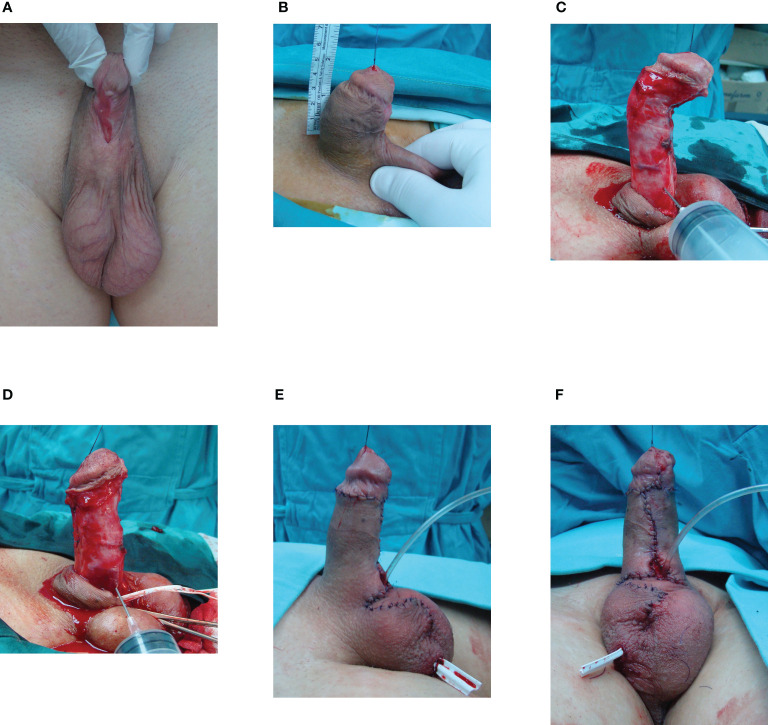
Cripple penis after multiple hypospadias surgeries in childhood. **(A)** Multiple scars with glans deformity and urethral defect. **(B)** Penis is 4cm long. **(C)** After degloving, artificial erection revealed ventral curvature. Scarred urethra is excised. **(D)** Correction of curvature is performed by dorsal plication. **(E)** Result after surgery. **(F)** Penile shaft is covered using skin flaps. Urethral reconstruction is left for the second stage.

**Figure 3:**
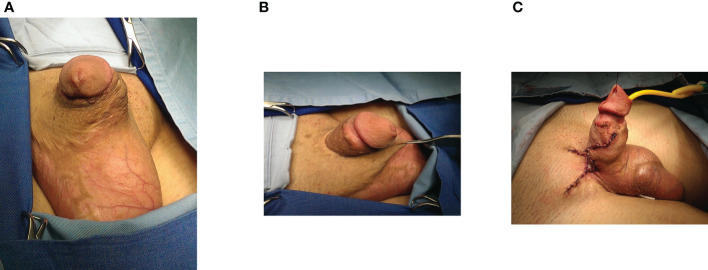
Reconstruction of trapped penis after failed hypospadias repair. **(A)** Penis is trapped after multiple failed surgeries in childhood. **(B)** Penile body trapped in insufficient, scared skin **(C)** Penile shaft is released and covered with local skin flaps for maximal lengthening.

Mobilization of the glans wings for glans reconstruction was performed in 30 cases. Radical glans dissection and reconstruction were performed to sculpture a conically shaped glans. (**Figure 1**) “Double-faced” skin flap was used for reconstruction in 7 cases. Penile curvature that required correction was confirmed intraoperatively in 48 cases (50%). There were 46 cases of residual ventral curvature, and 2 cases of lateral curvature. Plication of the tunica albuginea was done in 45 cases, as shown in **Figure 2**. The grafting technique was used in one lateral curvature and two cases of cripple penis to correct severe ventral curvature. In most trapped penis cases (29), vascularized skin flaps were created and used to cover the penile shaft. (**Figures 2**, **3**) Free skin grafts were used for reconstruction of the penile shaft in 4 cases of extremely insufficient healthy skin. Urethroplasty was performed in 25 patients, along with correction of other deformities. We used combined buccal mucosa graft and hairless skin flap, urethral plate and skin flap, urethral plate tubularization and urethral mobilization in 9, 8, 6 and 2 cases, respectively. (**Figures 1**, **2**)

Of the 97 patients, 83 (85.5%) underwent one-stage surgery for the correction of all deformities. Two-stage repair was performed in 14 patients (14.5%) with lack of available genital skin. The mean follow-up was 75 months (ranged from 24 to 168 months). Success was defined as a straight penis with good length, anatomically regular glans, and an overall cosmetically acceptable appearance, without the need for additional surgeries. The success rate of the procedure was 94%. Higher complication rates were observed after two-stage repair (14%) than after one-stage surgery (5%). Complications included 4 cases of penile curvature with late onset and one case of glans dehiscence, which were repaired 6 to 12 months after the previous surgery. Partial skin necrosis occurred in one patient who underwent a two-stage procedure and was resolved with conservative treatment. (**Table 3**)

**Table 3:** Outcomes of one-stage and two-stage repair.

Surgery	Number of patients (%)	Success rate	Re-curvature (%)	Glans dehiscence (%)	Partial skin necrosis (%)	Total (%)
**One-stage**	83 (85.5%)	95%	3 (3.5%)	1 (1%)	0	4 (5%)
**Two-stage**	14 (14.5%)	86%	1 (7%)	0	1 (7%)	2 (14%)
**Summary**	97 (100%)	94%	4 (4%)	1 (1%)	1 (1%)	6 (6%)

In the group of 25 patients who underwent simultaneous urethral reconstruction, we performed one-stage urethroplasty in 22 patients. Two-stage urethroplasty was performed in three patients. Complications included two cases of urethral fistula and one case of urethral dehiscence due to infection, repaired by minor surgery 6 months later.

Seventy-four patients completed the questionnaires postoperatively. Some degree of erectile dysfunction (IIEF-5 score<26) was reported in 24% (18/74) of the patients. Severe, moderate, and mild erectile dysfunction were reported in 6 (8%), 2 (3%) and 10 (13%) patients, respectively. All 18 patients were referred for further treatment of erectile dysfunction.

## Discussion

In the past, the main goal of hypospadias surgery was to bring the urethral meatus to the top of the glans. However, the concept and treatment of hypospadiac anomaly have changed. There is evidence that the surgeons and patients disagree about what produces a good surgical result, and many studies have shown that the patient’s satisfaction is the most relevant outcome measure (2, 9, 10). The main objective for the person with hypospadias is a normal penis with satisfactory sexual function, not the meatal position or micturition. Fichtner et al. evaluated a group of 500 men who underwent prostate surgery and identified 65 patients with hypospadiac meatus and without curvature (11). In this group, 60% of men and 55% of their partners were unaware of the existing anomaly. For a complete assessment of the hypospadias repair outcome, long-term follow up is imperative. Regular voiding and straight penis can be determined in childhood, but complete evaluation of results cannot be made until adulthood, when self-evaluation of sexual function is feasible. However, many of these patients are lost to follow-up in adolescence but can present in adulthood requiring surgical intervention. These patients wander between pediatric and adult urologists, which is an additional problem for them, emphasizing the need for transitional care (3, 12, 13).

Primary hypospadias repair may result in complications that occur in adolescence or even later. However, the nature, range, and appropriate treatment of these delayed complications remain poorly defined (14). In 126 patients who underwent one-stage hypospadias repair, Nuininga et al. reported a complication rate of 54% at long-term follow-up (15). The complication rate during the first 5 years was 37%, and 17% afterwards. In their study, the interval between surgery and the onset of complications was up to 14 years. Ching et al. described approximately 30% of patients with delayed complications after an initially successful repair in childhood (12). Analyzing 55 adults who underwent hypospadias repair, they classified this heterogeneous group as patients who underwent multiple surgeries (58%), patients with delayed complications (30%) and primary repairs (12%). In their cohort, persistent chordee was the third most common overall symptom (23.6%), after voiding symptoms (81.8%) and urinary tract infection (36.4%).

In our study, the mean time after primary repair of distal and proximal hypospadias is 15 and 19.5 years, respectively. Number of previous surgeries and severity of hypospadias are considered major risk factors for poor outcomes (2). Conversely, most of our adult patients with non-urethral complications (65%) initially presented with distal hypospadias, as shown in **Table 1**. Residual curvature is the most common non-urethral complication in this group, especially in distal hypospadias (50% of all complications). During primary repair, we found that almost 25% of distal hypospadias were associated with significant curvature (16). Penile curvature can have a long-term impact on multiple levels, including voiding, sexual activities and subjective genital image. Therefore, intraoperative diagnosis of associated curvature should not be reserved exclusively for severe hypospadias. Another important issue is the trapped penis after previous surgeries, due to scarring and lack of quality penile skin. This entity has been well defined in children but is poorly evaluated in adults (17). In our study, it is the most common late complication of proximal hypospadias repair, occurring in 50% of proximal cases treated, as presented in **Table 2**. This leads to penile shortening, which has already been reported as one of the patients’ main complaints and problems (6).

Surgical treatment of late complications after hypospadias repair has not been standardized. Preoperative assessment of all deformities and a detailed surgical plan are necessary for a successful outcome. Most authors prefer staged procedures, but it is commonly understood that each patient should be evaluated separately, and the treatment should be individualized and preferably conducted in a specialized center, with experience in transitional urology (18–20). In our study, a radical approach was used in all cases to correct all associated complications. One-stage repair was performed in most patients (85.5%), with a success rate of 95%. In cases where a two-stage surgery was necessary, a successful outcome was achieved in 86% of the cases. Therefore, we suggest correction of all deformities in one stage, when possible, as presented in **Figure 1**. Nevertheless, correction of residual curvature and achieving good penile length and appearance should be an imperative, and it seems reasonable in those cases to leave urethroplasty for the next stage, as presented in **Figure 2**.

The psychosexual aspect, including erectile function, is reported to be generally affected in patients with hypospadias, mostly in proximal forms (1, 4, 6). Our study indicates a significant number of adult patients with erectile dysfunction after hypospadias surgery in childhood (24%). However, an obvious limitation of this study is the lack of a control group and preoperative assessment of sexual function, which would provide the most objective evaluation. Another limitation of the research is that the majority of patients were initially treated elsewhere, with missing reports in 12 cases and possibly misleading patient-reported data.

Non-urethral complications may occur many years after primary hypospadias repair and have a strong impact on quality of life. Surgical treatment is individualized, and usually involves a radical approach and complex reconstruction to correct all the presenting deformities and achieve successful cosmetic and psychosexual outcome. The key to a successful outcome in hypospadias is long-term follow-up, not only until adolescence but also in adulthood.

## Data availability statement

The original contributions presented in the study are included in the article/supplementary material. Further inquiries can be directed to the corresponding author.

## Ethics statement

Written informed consent was obtained from the individual(s) for the publication of any potentially identifiable images or data included in this article.

## Author contributions

BS and MD contributed to conception and design of the study. BS and MBi organized the database. MBi and MBe performed the statistical analysis. BS wrote the first draft of the manuscript. MBi and MBe wrote sections of the manuscript.
